# Visual Field Defects and Retinal Nerve Fiber Layer Thickness in Migraine Patients: A Study From a Tertiary Care Center in India

**DOI:** 10.7759/cureus.66342

**Published:** 2024-08-06

**Authors:** Raja AM, Seema G, Praveena Daya A, Shanmugavinayagam Arumuganathan, Rajeswari Kathiah, Sarah Ramamurthy

**Affiliations:** 1 Department of Ophthalmology, All India Institute of Medical Sciences, Madurai, Tamil Nadu, IND; 2 Department of Community and Family Medicine, All India Institute of Medical Sciences, Madurai, Tamil Nadu, IND; 3 Department of Psychiatry, All India Institute of Medical Sciences, Madurai, Tamil Nadu, IND; 4 Department of Pathology, All India Institute of Medical Sciences, Madurai, Tamil Nadu, IND; 5 Department of Anatomy, All India Institute of Medical Sciences, Madurai, Tamil Nadu, IND

**Keywords:** optical coherence tomography (oct), retinal nerve fiber layer, headache, visual field defects, migraine

## Abstract

Background

This study aimed to analyze the visual field changes and retinal nerve fiber layer (RNFL) thickness during the headache phase of migraine attacks among migraine patients compared with controls.

Methodology

A prospective, case-control study was conducted at a tertiary care center in Palakkad, Kerala from January 2022 to August 2023. This study included 50 migraine patients and 50 age/gender-matched controls. Adults aged 20-40 years with a more than three-year history of migraine were included in this study and those who had systemic or ocular pathologies were excluded. All 100 subjects underwent complete ocular examination, including full threshold 24-2 automated perimetry for visual field analysis and optical coherence tomography for analyzing RNFL thickness. Statistical analysis was done using SPSS Statistics Version 25 (IBM Corp., Armonk, NY, USA).

Results

In this study, the average age for cases was 29.24 ± 5.10 years, and for controls was 30.12 ± 6.20 years. Gender distribution was identical between cases and controls with 29 (58%) females and 21 (42%) males. Among the 50 migraine patients, 22 (44%) had generalized, while 28 (56%) had localized field defects during the headache phase of migraine attacks. There was a statistically significant (p < 0.001) difference in superior quadrant RNFL thickness between cases (114.08 ± 12.25) and controls.

Conclusions

We found that RNFL thinning in the superior quadrant and non-specific localized visual field changes occur during migraine attacks. We conducted this study in a tertiary care center as very few studies in our country have revealed visual field changes during migraine headache attacks.

## Introduction

Migraine, a complex neurological condition characterized by recurrent headaches and various sensory disturbances, affects a significant proportion of the global population [1]. Beyond its hallmark symptom of severe headaches, migraines can encompass transient neurological symptoms known as aura, often involving visual disturbances such as flashes of light, blind spots, or zigzag lines [2]. These manifestations underscore the intricate interplay between vascular dysregulation, neuronal hyperexcitability, and genetic predisposition that underpins the pathophysiology of migraine [1,2].

While the primary focus of migraine research has traditionally centered on headaches and associated symptoms, recent studies have increasingly explored its extracranial manifestations, particularly its impact on the visual system [3]. Visual disturbances during migraine attacks, including visual field defects and alterations in retinal nerve fiber layer (RNFL) thickness, have garnered attention due to their potential implications for visual health and quality of life [4].

The RNFL, consisting of axons from retinal ganglion cells, serves as a critical component of the optic nerve and is essential for visual function. Changes in RNFL thickness, detectable through advanced imaging techniques such as optical coherence tomography (OCT), may reflect neurodegenerative processes or acute ischemic events affecting the retina and optic nerve. Understanding these changes could provide valuable insights into the broader neurological implications of migraine beyond the headache phase [3,5].

Despite growing recognition of the link between migraine and visual disturbances, comprehensive studies specifically investigating visual field defects and RNFL thickness in migraineurs remain relatively scarce. Addressing this gap is crucial not only for advancing scientific understanding but also for improving clinical management strategies [2,6]. By elucidating the prevalence, characteristics, and potential mechanisms underlying visual changes in migraine patients, this study aims to contribute to a deeper understanding of migraine as a systemic neurovascular disorder with significant ocular manifestations.

## Materials and methods

This prospective, case-control study was conducted at a tertiary care center in Palakkad, Kerala from January 2022 to August 2023. A total of 50 migraine patients and 50 controls were included in this study after obtaining Institutional Ethical Committee approval from the Government Medical College, Palakkad (approval number: GMC/PKD/IEC/01/2022/52).

Study participants were recruited based on specific criteria. Inclusion criteria for cases included adults aged 20 to 40 years with a history of migraine, whether with aura or without, lasting more than three years, as diagnosed through clinical examination. Exclusion criteria for cases encompassed individuals with other types of headaches such as sinusitis or cluster headaches, systemic diseases such as diabetes mellitus (DM), systemic hypertension (SHT), or intracranial space-occupying lesions, as well as ocular conditions such as refractive errors, presbyopia, glaucoma, and papilledema. Controls were selected based on the inclusion criteria of adults aged 20 to 40 years with no history of migraine within the past 12 months. Exclusion criteria for controls mirrored those for cases, specifically excluding individuals with other types of headaches such as sinusitis or cluster headaches, systemic diseases such as DM or SHT, and ocular diseases such as refractive errors, presbyopia, glaucoma, and papilledema.

All 50 migraine patients were diagnosed and referred to the ophthalmology outpatient department (OPD) by a neurologist based on the International Classification of Headache Disorders guidelines. Age/gender-matched controls were randomly selected from the ophthalmology department.

The postgraduate residents were trained to explain the OCT and visual field analysis and sensitize all the study participants to report to the ophthalmology OPD within one to two hours following the headache phase of migraine attacks. Initially, at the first visit, when they were symptom-free, written informed consent was obtained in the form of a Declaration of Helsinki from all participants who fulfilled the inclusion criteria. A semi-structured proforma was administered to obtain demographic details of the participants such as age, sex, and occupation; clinical characteristics such as the duration of migraine, the number of attacks/month, the side of involvement; and associated precipitating factors and autonomic symptoms. Both cases and controls underwent the following ocular examinations: visual acuity using Snellen’s chart, slit lamp examinations using Topcon model slit lamp, intraocular pressure (IOP) of both eyes measured by Goldman applanation tonometer, fundus examination by +90 D lens, and indirect ophthalmoscopy.

During the headache phase of migraine, all cases underwent visual field analysis and RNFL measurements within one to two hours of presentation. Using Humphrey’s Field Analyzer (HFA II 750, Carl Zeiss Meditec, USA), a full threshold central 24-2 Static Automated Perimetry (SAP) was exclusively done on the eye affected by migraine symptoms. Global indices such as mean deviation (MD), pattern standard deviation (PSD), corrected pattern standard deviation (CPSD), and glaucoma hemifield test (GHT) results were recorded. The visual field was abnormal if the GHT was outside the normal limits ± presence of a cluster of ≥three depressed non-edge points in the field chart. Perimetry values were non-reliable if fixation loss was >20% and false-positive (or) false-negative responses were >33%. The eye on the side of the headache during migraine attacks was subjected to three circular scans around the center of the optic disc with signal strength greater than seven after pupillary dilatation. The mean of the three scans provided the superior, inferior, temporal, and nasal parts of RNFL thickness. RNFL thickness was measured with a Cirrus OCT machine (Cirrus OCT 500, Carl Zeiss Meditec, USA). RNFL thickness was differentiated from other retinal layers using an edge detection algorithm.

Data were collected in a Microsoft Excel sheet 2016 (Microsoft Corp., Redmond, WA, USA) and were analyzed using SPSS Statistics Version 25 (IBM Corp., Armonk, NY, USA). Categorical variables such as gender were expressed as frequency and percentages, and continuous variables such as age, IOP, duration of migraine, frequency of migraine, and RNFL thickness were expressed as mean and standard deviation (SD) as the variables followed a normal distribution. To compare the means between cases and controls, an independent samples t-test was used, and a p-value less than 0.05 was considered statistically significant for all comparisons.

## Results

In this study, a cohort of 100 individuals were enrolled, comprising 50 migraine cases and 50 controls, after carefully matching for age and gender.

**Figure 1:**
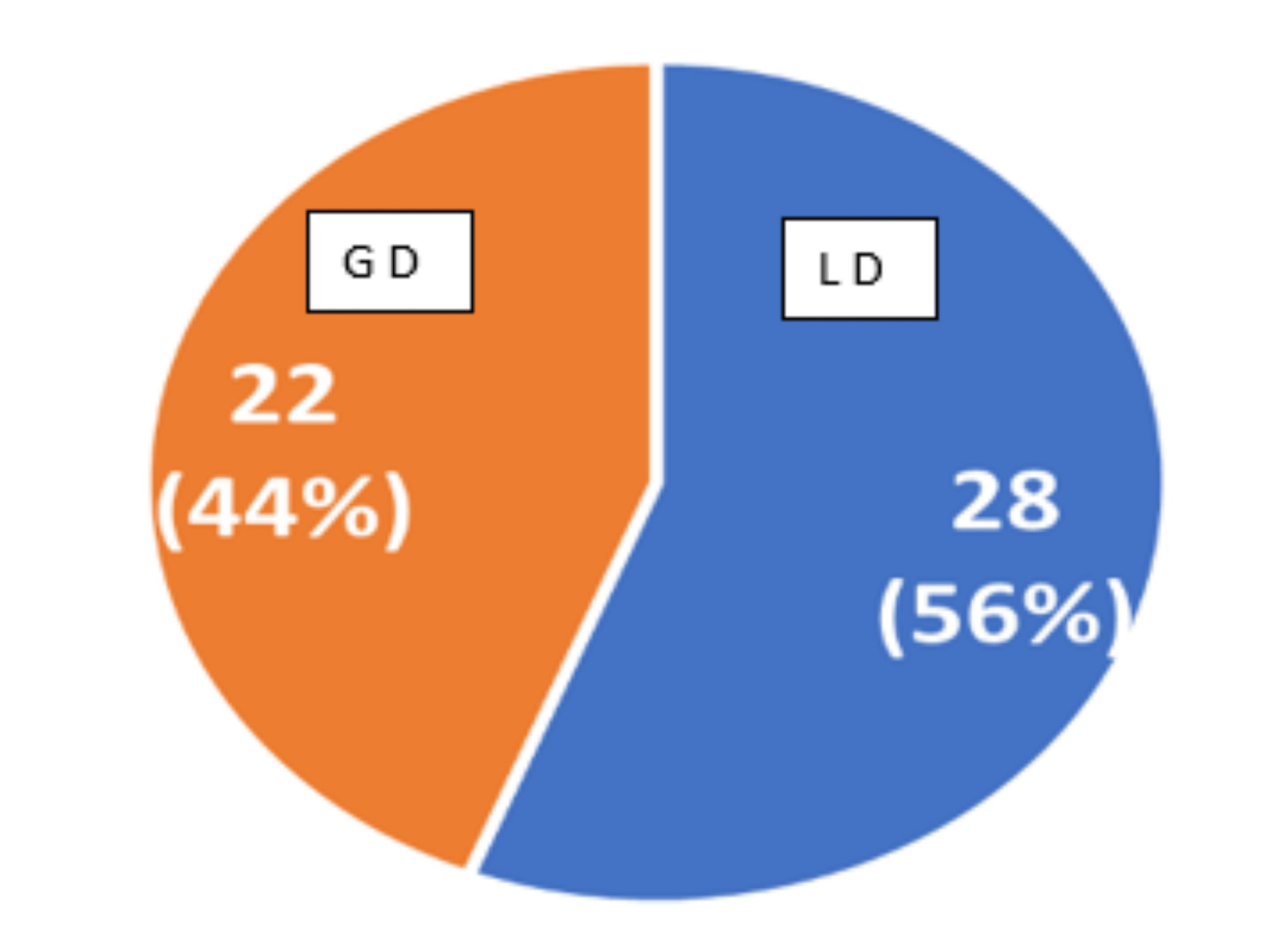
Visual field defects among patients with migraine (n = 50). GD: generalized visual field defect; LD: localized visual field defect

Table 1 presents the demographic and clinical profile of the study participants. The average (mean ± SD) age for cases and controls was 29.24 ± 5.10 and 30.12 ± 6.2, respectively. Gender distribution revealed that among the cases, 29 (58%) were females and 21 (42%) were males, while in the control group, the proportions were identical with 29 (58%) females and 21 (42%) males. The mean ± SD of IOP was 15.96 ± 2.27 among the cases and 15.48 ± 2.20 among the controls, indicating a non-significant difference between the two groups. In our study, 46% of patients had predominantly migraine involvement on the right side and 54% had left-side involvement. Among the 50 cases of migraine patients, 22 (44%) had generalized field defects, while 28 (56%) presented localized field defects. None of the participants in the control group displayed visual field defects (Figure 1).

**Table 1:** Demographic and clinical characteristics among participants (n = 100). *: Age in years and intraocular pressure (IOP) in mmHg are expressed as mean ± SD. **: Distribution of gender is expressed as n (%). ***: A p-value less than 0.05 is considered statistically significant.

Variables	Statistics	Cases (N = 50)	Controls (N = 50)	P-value^***^
Age^*^	Mean ± SD	29.24 ± 5.10	30.12 ± 6.20	1.00
IOP^*^	Mean ± SD	15.96 ± 2.27	15.48 ± 2.20	0.40
Gender^**^	Females	29 (58%)	29 (58%)	1.00
	Males	21 (42%)	21 (42%)	

Table 2 illustrates the distribution of the duration of migraine in years and the frequency of migraine attacks among both cases and controls. Among the migraine cases, the average duration of migraine in years was 6.84 ± 3.18, while the frequency of migraine attacks per month was 4.60 ± 1.64.

**Table 2:** Duration of migraine and frequency of migraine among study participants (n = 100). *: Duration of migraine is expressed as years and frequency of migraine attacks is expressed as mean ± SD with a 95% confidence interval (CI).

Statistics	Case/Control	Mean ± SD	Minimum, maximum	Mean difference (95% CI)
Duration of migraine in years^*^	Cases	6.84 ± 3.18	2, 15	6.84 (5.93, 7.74)
Frequency of migraine attacks/month*	Cases	4.60 ± 1.64	2, 8	4.60 (4.13, 5.06)

Table 3 presents the mean and SD of RNFL thickness for both cases and controls. A statistically significant difference in RNFL thickness was observed between the two groups, with cases exhibiting thinner RNFL compared to controls in the superior quadrant.

**Table 3:** Comparison of the mean RNFL between migraine patients and controls (n = 100). *: Retinal nerve fiber layer (RNFL) thickness is expressed as mean ± SD with a 95% confidence interval (CI). **: A p-value less than 0.05 is considered statistically significant.

RNFL thickness^*^	Case/Control	N	Mean ± SD	Mean. diff. (95% CI)	t value	P-value**
Superior	Case	50	114.08 ± 12.25	-13.12 (-19.48, -6.75)	-1.27	<0.001
	Control	50	127.20 ± 19.08			
Inferior	Case	50	135.16 ± 17.21	2.52 (-4.94, 9.98)	0.67	0.17
	Control	50	132.64 ± 20.25			
Temporal	Case	50	68.26 ± 12.83	1.06 (-4.13, 6.25)	0.40	0.49
	Control	50	67.20 ± 13.31			
Nasal	Case	50	83.44 ± 16.17	-3.30 (-3.33, 9.93)	0.98	0.37
	Control	50	80.14 ± 17.22			

## Discussion

This cross-sectional study analyzed visual field defects and RNFL thickness during the headache phase of migraine attacks in migraine patients which was compared with age and gender-matched controls. In our study, the average age was 29. 24 ± 5.10 for the migraine patients. Among the cases, 29 (58%) were females and 21 (42%) were males, identical with the control group. The mean ± SD of IOP was 15.96 ± 2.27 among the cases and 15.48 ± 2.20 among the controls. In our study, 46% of patients had migraine involvement predominantly on the right side and 54% had it on the left side. Peripheral contraction of the visual field was seen in most migraine patients during migraine attacks. Localized visual field changes (56%) were more common than generalized visual field changes (44%). We noted that 46% of patients had migraine involvement predominantly on the right side and 54% had left-side involvement, which was similar to the study by Salman et al. [5].

Lipton et al. suggested the peak age for migraine incidence was 30 to 40 years and declined after 60 years [6-9]. In our study, the majority of the subjects who presented with migraine symptoms were females (58%) compared to males (42%), which is similar to the World Health Organization reports [2]. Our study revealed that the average duration of migraine was 6.84 ± 3.18 years and the frequency of migraine attacks per month was 4.60 ± 1.64. During a migraine attack, the eye corresponding to the affected side was assessed; the right eye was examined for 23 patients, and the left eye was examined for 27 patients. The mean ± SD of IOP was 15.96 ± 2.27 mmHg for cases and 15.48 ± 2.20 for controls, and the difference was not statistically significant. Similar findings were reported by Dersu et al. [6,7].

In this study, we found that peripheral contraction of the visual field was seen in most migraine patients during migraine attacks. Localized visual field changes (56%) were more common than generalized visual field changes (44%). However, localized field changes were non-specific, unilateral, and non-homonymous which correlated with the study conducted by Salman et al. [5]. Lewis et al. found that migraine subjects have visual field changes in the form of generalized depression rather than localized changes [10,11]. During migraine attacks, activation of the trigeminovascular system and release of neuropeptides leads to cerebral vasospasm, which may affect ocular perfusion to the anterior optic nerve head and cause retinal ganglion cell death [12]. Perry et al. suggested that retinal ganglion cells have magnocellular or bistratified (20%) ganglion cells and parvocellular (80%) ganglion cells [13]. Short wavelength automated perimetry or blue-on-yellow perimetry is more sensitive in detecting early visual field changes in migraine than SAP through the S-core neuronal pathway from bistratified ganglion cells [14,15], which was confirmed by Kendrick et al. Overall, 50% (n = 25) of migraine subjects were randomly selected and subjected to visual field analysis and OCT five hours from the time of onset of headache. Visual field changes and RNFL thickness completely normalized after the migraine attacks [5]. There was no correlation with age, sex, duration, frequency, and side of migraine, and the findings correlated with the study by Salman et al. [5]. However, Lewis et al. found that the prevalence of visual field loss was greater with increasing age and duration of the disease [11]. The visual field was normal for all controls.

This study showed a statistically significant difference in RNFL thickness in the superior quadrant between migraine cases and controls. The mean ± SD among cases was 114.08 ± 12.25 during a migraine attack and among controls was 127.20 ± 19.08, and these findings correlated with the study by Michael et al. [7], Gipponi et al., and Kirbas et al. [16-18]. Tan et al. found no discernible variance in RNFL thickness between migraine patients and healthy groups [16]. The differences observed between the findings of the current study and those of Tan et al. can be attributed to methodological disparities. Tan et al. employed scanning laser polarimetry for assessing the RNFL thickness, whereas the current study utilized Cirrus HD OCT.

In the study by Martinez et al., RNFL thickness was measured using OCT and found no significant difference in RNFL thickness between migraine cases and controls [19]. Studies have shown inconsistent RNFL thickness results among migraine cases. This can be attributed to different measurement methods, sample size, lack of an international standardized format for inclusion/exclusion criteria, and racial differences. Many studies have revealed normal RNFL or thinning in individual quadrants or thinning in mean RNFL [2]. Corbett et al. found a higher prevalence of migraine with low-tension glaucoma [20-22] and suggested migraine can be considered a potential risk factor for developing low-tension glaucoma which was confirmed by the Blue Mountain Eye study, one of the large population studies [23]. During migraine attacks, vasospasms of cerebral and retrobulbar vessels are a transient phenomenon but chronicity of the disease is believed to be a risk factor for structural damage to the brain and optic nerve head. Newer technologies such as transcranial Doppler and OCT angiography can be used to study changes in cerebral and ocular blood flow and resultant structural changes in the thickness and vasculature of select retinal layers and choroid in migraine patients, thereby providing additional clues to confirm the presence of a common vascular etiology in both migraine and glaucoma [8]. Performing routine fundus examinations for all migraine patients can aid in the early detection of glaucoma and facilitate prompt initiation of treatment. 

The small sample size and the lack of assessment of optic nerve head parameters are the major limitations of this study. The study also noted that ocular changes completely disappeared after migraine attacks, but the exact timing and duration of these changes were not extensively explored. Understanding the temporal dynamics could provide deeper insights into the pathophysiology.

## Conclusions

In our study, we found that RNFL thinning in the superior quadrant and non-specific localized visual field changes occur during migraine attacks. Very few studies have revealed visual field changes during the headache phase of migraine attacks in India. As migraine headache is the most common health issue in adults, it affects patients’ quality of life and causes productivity losses to society. Further investigations are warranted to identify whether the observed visual field changes stem from cortical, retinal, or vascular origins. Migraines require considerable attention from public health systems for awareness campaigns, research funding, and the development of better treatment protocols.

## References

[1] Amer SA, Hassan MA, Saif YS, Samir H (2006). Headache with ophthalmological correlation in children and young adolescents a study of 50 cases. Bull Ophthalmol Soc Egypt.

[2] World Health Organization (2011). Atlas of Headache Disorders and Resources in the World. https://iris.who.int/bitstream/handle/10665/44571/9789241564212_eng.pdf.

[3] (2013). The International Classification of Headache Disorders, 3rd edition (beta version). Cephalalgia.

[4] Ashina S, Bendtsen L, Ashina M (2012). Pathophysiology of migraine and tension-type headache. Tech Reg Anesth Pain Manage.

[5] Salman AG, Hamid MA, Mansour DE (2015). Correlation of visual field defects and optical coherence tomography finding in migraine patients. Saudi J Ophthalmol.

[6] Dersu II, Thostenson J, Durcan FJ, Hamilton SM, Digre KB (2013). Optic disc and visual test findings in patients with migraine. J Clin Neurosci.

[7] Michael ND, Hussein A, Abd Halim S, Ab Hamid SA (2019). Evaluation of optic nerve head parameters, retinal nerve fiber layer thickness, and ocular perfusion pressure in migraine patients. Cureus.

[8] Panicker G, Kaliaperumal S, Narayan S, Mani M (2021). Glaucoma and optical coherence tomography changes in migraine: a comparative cross-sectional study. Indian J Ophthalmol.

[9] Lipton RB, Bigal ME, Diamond M, Freitag F, Reed ML, Stewart WF (2007). Migraine prevalence, disease burden, and the need for preventive therapy. Neurology.

[10] McKendrick AM, Vingrys AJ, Badcock DR, Heywood JT (2000). Visual field losses in subjects with migraine headaches. Invest Ophthalmol Vis Sci.

[11] Lewis RA, Vijayan N, Watson C, Keltner J, Johnson CA (1989). Visual field loss in migraine. Ophthalmology.

[12] Russo A, Tessitore A, Tedeschi G (2013). Migraine and trigeminal system-I can feel it coming…. Curr Pain Headache Rep.

[13] Perry VH, Oehler R, Cowey A (1984). Retinal ganglion cells that project to the dorsal lateral geniculate nucleus in the macaque monkey. Neuroscience.

[14] McKendrick AM, Cioffi GA, Johnson CA (2002). Short-wavelength sensitivity deficits in patients with migraine. Arch Ophthalmol.

[15] Yenice O, Temel A, Incili B, Tuncer N (2006). Short-wavelength automated perimetry in patients with migraine. Graefes Arch Clin Exp Ophthalmol.

[16] Tan FU, Akarsu C, Güllü R (2005). Retinal nerve fiber layer thickness is unaffected in migraine patients. Acta Neurol Scand.

[17] Gipponi S, Scaroni N, Venturelli E (2013). Reduction in retinal nerve fiber layer thickness in migraine patients. Neurol Sci.

[18] Kirbas S, Tufekci A, Turkyilmaz K, Kirbas A, Oner V, Durmus M (2013). Evaluation of the retinal changes in patients with chronic migraine. Acta Neurol Belg.

[19] Martinez A, Proupim N, Sanchez M (2008). Retinal nerve fibre layer thickness measurements using optical coherence tomography in migraine patients. Br J Ophthalmol.

[20] Sorkhabi R, Mostafaei S, Ahoor M, Talebi M (2013). Evaluation of retinal nerve fiber layer thickness in migraine. Iran J Neurol.

[21] Feng YF, Guo H, Huang JH, Yu JG, Yuan F (2016). Retinal nerve fiber layer thickness changes in migraine: a meta-analysis of case-control studies. Curr Eye Res.

[22] Corbett JJ, Phelps CD, Eslinger P, Montague PR (1985). The neurologic evaluation of patients with low-tension glaucoma. Invest Ophthalmol Vis Sci.

[23] Wang JJ, Mitchell P, Smith W (1997). Is there an association between migraine headache and open-angle glaucoma? Findings from the Blue Mountains Eye Study. Ophthalmology.

